# Digital nudging in software development: a review and research agenda

**DOI:** 10.3389/frai.2026.1898557

**Published:** 2026-09-01

**Authors:** Ingo Pribik, Alexander Felfernig

**Affiliations:** Institute of Software Technology, Graz University of Technology, Graz, Austria

**Keywords:** AI-assisted development, cognitive biases, digital nudging, persuasive technology, software engineering, software quality, SOLID principles

## Abstract

Digital nudging, a behavioral approach that subtly guides decision-making, is gaining increasing attention in software development as a means to support developers in complex and cognitively demanding tasks. This article analyzes the evolution of digital nudging and persuasive technology in software engineering from 2010 to April 2026. Based on a structured literature review, a temporal heatmap and qualitative trend analysis are used to examine how established nudging strategies, such as feedback, framing, and personalization, have been applied to support software development activities. The results demonstrate a shift from rule-based and socially oriented interventions toward more adaptive, context-aware, and AI/LLM-mediated approaches. In particular, recent work shows an increasing integration of personalized and real-time nudging mechanisms directly into development environments. Building on these findings, the article introduces a harmonized perspective on digital nudging, in which multiple strategies are systematically integrated to provide consistent and context-aware decision support. An illustrative AI-driven plug-in prototype demonstrates how personalized nudging mechanisms can be integrated into a development environment to support software quality and adherence to design principles such as SOLID. Furthermore, key challenges are identified, including the selection and combination of nudging strategies, their evaluation, and their integration into real-world development workflows. Overall, the article contributes a structured synthesis of existing research and outlines directions for future work on harmonized, AI-driven nudging in software engineering.

## 1 Introduction

Software development teams frequently operate under significant pressure to deliver high-quality solutions within limited time frames (Basten et al., 2021). At the same time, ongoing digitalization, the growth of data-intensive systems, the adoption of new technology stacks, and continuously evolving business requirements contribute to increasing complexity in software projects (Hummel et al., 2018; Salamah and Alnaji, 2014). These conditions can introduce instability and increase uncertainty throughout the development lifecycle.

Empirical studies indicate that many software projects encounter substantial challenges. For example, the **Standish Group's 2018 CHAOS Report**, based on the analysis of more than 50,000 IT projects, reports that between 64% and 86% of projects are categorized as “challenged” or “failed,” depending on the applied success criteria (The Standish Group, 2018). While such classifications depend on how success is defined, the reported outcomes point to persistent challenges in delivering software projects on time, within budget, and in alignment with stakeholder expectations.

Process-related factors play an important role in project outcomes. However, outcomes are also influenced by the day-to-day decision-making practices of software engineers. Software development and architecture involve numerous design decisions that shape a system's structure and long-term evolution. As Medvidovic and Taylor (2010) describe, software architecture consists of the principal design decisions made during a system's development and evolution. Consequently, early and recurring design decisions may have lasting effects on maintainability, extensibility, and overall system evolution. These decisions are often guided by established design principles, such as the **SOLID** principles (Martin, 2003), which aim to improve modularity and adaptability at the code and component level.

In addition to technical considerations, decision-making in software development is shaped by cognitive mechanisms that influence how individuals interpret information, recall prior experiences, generate alternatives, and evaluate trade-offs. Empirical research suggests that cognitive biases are observable in development contexts. For instance, Chattopadhyay et al. (2022) report that a substantial share of observed developer behaviors can be associated with cognitive bias. Under conditions such as time pressure or uncertainty, developers may rely on familiar solutions, postpone refactoring, or overlook alternative design options. These behaviors can be understood as systematic cognitive tendencies rather than deficiencies in expertise.

Given these behavioral influences, research has increasingly explored approaches that support decision-making without imposing rigid constraints. In this context, persuasive technology and digital nudging have gained attention in both behavioral science and software engineering research (Caraban et al., 2019; Thaler and Sunstein, 2008). Persuasive technology refers to interactive systems designed to influence users' attitudes or behaviors (Fogg, 2003). Digital nudging, which is central to this article, focuses on structuring decision environments in ways that guide choices while preserving individuals' freedom of choice.

Building on this perspective, this article provides a structured overview of digital nudging in software development. Specifically, it:

1. presents an overview of nudging types discussed in the literature published from 2010 to April 2026 in the context of software development,
2. discusses psychological effects relevant to software development, with attention to different development phases,
3. identifies examples of tools that incorporate digital nudging mechanisms,
4. examines open issues and challenges associated with applying nudging mechanisms in software development, and
5. introduces a harmonized perspective on digital nudging and illustrates its application through an AI-driven plug-in prototype designed to support adherence to software design principles such as **SOLID**.

The remainder of this article is structured as follows. Section 2 provides background on software design principles, cognitive biases, persuasive technology, and nudging theory. Section 3 describes the methodology and literature search process. Section 4 examines the evolution of digital nudging in software development through a temporal analysis. Section 5 introduces the concept of harmonized digital nudging and outlines key design dimensions. Section 6 presents an illustrative example of an AI-assisted nudging approach for supporting SOLID compliance. Section 7 discusses open research challenges, and Section 8 concludes the article.

## 2 Background

### 2.1 SOLID principles in software design

The acronym **SOLID** refers to a set of five widely recognized principles in object-oriented design (Martin, 2003): **Single Responsibility**, **Open/Closed**, **Liskov Substitution**, **Interface Segregation**, and **Dependency Inversion**. Together, these principles provide general guidelines for structuring software systems in a way that supports clarity and long-term sustainability.

Rather than prescribing specific implementation techniques, the **SOLID** principles offer a conceptual framework for making design decisions that improve the internal organization of software. They encourage developers to decompose systems into well-defined components, establish clear abstractions, and reduce unnecessary coupling between parts of a system. As a result, systems designed with these principles in mind tend to be easier to understand, modify, and extend over time.

The **Single Responsibility Principle (SRP)** states that a class should focus on one primary responsibility, thereby improving modularity, clarity, and maintainability (Martin, 2003). For example, an Employee class should primarily manage employee-related information rather than simultaneously handling payroll calculations or database persistence.

The **Open/Closed Principle (OCP)** suggests that software components should be open for extension but closed for modification, allowing new functionality to be introduced without changing existing implementations (Meyer, 1988). For instance, additional employee types such as ManagerEmployee or RemoteEmployee can extend existing functionality without requiring modifications to the original Employee class.

The **Liskov Substitution Principle (LSP)** states that derived classes should remain interchangeable with their base classes without affecting expected program behavior (Liskov, 1988). A specialized ManagerEmployee class should therefore behave consistently wherever an Employee object is expected.

The **Interface Segregation Principle (ISP)** recommends the use of small and focused interfaces so that classes are not forced to implement functionality they do not require. Instead of requiring all employee-related classes to implement methods such as manageTeam(), only management-related classes should provide such functionality.

Finally, the **Dependency Inversion Principle (DIP)** states that high-level modules should depend on abstractions rather than concrete implementations. For example, an EmployeeService should depend on an abstract EmployeeRepository interface instead of directly relying on a specific database implementation.

Collectively, the **SOLID** principles support maintainability, flexibility, and extensibility. Their application depends on developers' day-to-day design decisions.

### 2.2 Cognitive biases in software development

Software development involves continuous decisions that shape the structure, evolution, and quality of software artifacts (Gall et al., 2018). These decisions may be influenced by cognitive biases, defined as systematic deviations from rational judgment (Tversky and Kahneman, 1974) that can affect decision quality and problem solving (Arnott, 2006).

Such influences arise across software engineering practice. Developers may favor short-term convenience when deciding whether to retain or replace an existing framework (Becker et al., 2019), adopt online code snippets without fully considering security implications (Fischer and Grossklags, 2022), deviate from recommended practices under time pressure (Mayer-Dorn et al., 2021), or be constrained by limited awareness of available software quality tools and practices (Murphy-Hill, 2012). Empirical evidence further demonstrates the presence of cognitive biases in developer behavior (Chattopadhyay et al., 2022).

The present work focuses on a subset of cognitive biases that are frequently discussed in both software engineering and decision-making literature and that are particularly relevant to software design decisions. These biases are summarized in Table 1.

**Table 1:** Selected cognitive biases relevant to software development and design decision-making.

Bias	Description	Relevance in software development
Anchoring bias	Tendency to rely heavily on initial information or decisions	Developers may adhere to early architectural choices or initial implementations even when alternative solutions would better support maintainability or extensibility
Confirmation bias	Favoring information that confirms existing beliefs while disregarding contradictory evidence	Developers may overlook indicators that suggest the need for refactoring or alternative design approaches
Overconfidence bias	Overestimating one's own knowledge, skills, or judgment	Developers may underestimate implementation risks or overestimate the robustness and quality of their designs
Availability bias	Preference for information that is easily recalled or familiar	Developers may repeatedly use known frameworks, tools, or patterns rather than evaluating potentially more suitable alternatives
Status quo bias	Preference for maintaining the current state of a system	Existing structures, dependencies, or architectural decisions may be retained despite evidence supporting improvement or modernization

Such effects are particularly relevant to software architecture and design, where decisions can have long-term consequences for maintainability, extensibility, and system quality. As software architecture can be understood as a set of design decisions that shape system evolution (Medvidovic and Taylor, 2010), cognitive biases may influence how developers evaluate alternatives and perceive existing solutions.

To better understand these effects, prior work has systematically investigated cognitive biases in software engineering. For example, Mohanani et al. (2020) identified a broad range of biases affecting software development activities through a systematic literature review. Building on this foundation, Haki et al. (2022) explored how psychological phenomena can be leveraged to design interventions that support more effective developer decision-making.

Given that cognitive biases may systematically influence software development decisions, there is a need for approaches that help developers make better choices without imposing rigid constraints. In this context, persuasive technology and digital nudging offer mechanisms for structuring decision environments in ways that encourage desirable behavior while preserving developer autonomy. These concepts are discussed in the following subsections.

### 2.3 Persuasive technology

Persuasive technology has been applied across a range of domains, including health and workplace environments, to influence user behavior and attitudes (Fogg, 2003; Oinas-Kukkonen and Harjumaa, 2009). According to Fogg (2003), persuasive technology refers to “computing systems, devices, or applications intentionally designed to change a person's attitudes or behavior in a predetermined way.” Unlike coercive approaches, persuasive technologies aim to encourage behavioral change while preserving users' freedom of choice.

The effectiveness of persuasive technology has been investigated across a variety of application domains. For example, Aldenaini et al. (2020) reviewed 170 studies examining the use of persuasive strategies to reduce sedentary behavior. These studies demonstrate the broad applicability of persuasive approaches across different contexts and user populations. Early applications in software engineering explored similar ideas by using persuasive mechanisms to encourage quality-oriented development behavior and support developers in making design decisions aligned with established quality principles (Pribik and Felfernig, 2012). These studies suggest that behavioral influence concepts can also be applied within software development environments.

To support the design of such systems, several frameworks have been proposed. One widely used approach is the **Persuasive Systems Design (PSD)** model proposed by Oinas-Kukkonen and Harjumaa (2009), which builds on earlier work in persuasive technology, including the **Fogg Behavior Model (FBM)** introduced by Fogg (2009). The **FBM** proposes that a target behavior occurs when three elements converge simultaneously: sufficient motivation, adequate ability to perform the behavior, and a prompt that triggers the action. According to the model, successful persuasive interventions should therefore increase users' motivation, reduce the effort required to engage in the desired behavior, and provide appropriate prompts at the right moment. Building on these principles, the **PSD** model provides a structured method for analyzing, designing, and evaluating persuasive systems and emphasizes the importance of context, system characteristics, and interaction design in shaping user behavior.

The **PSD** model organizes persuasive design principles into four categories: *Primary Task Support, Dialogue Support, System Credibility Support*, and *Social Support*. These categories describe different ways in which systems can assist and influence users. In total, the **PSD** framework comprises 28 persuasive design principles distributed across these four categories (Oinas-Kukkonen and Harjumaa, 2009).

Several *Primary Task Support* principles, such as reduction, tailoring, personalization, and self-monitoring, exhibit conceptual similarities to mechanisms that later emerged in the digital nudging literature. The primary task support principles most relevant to software engineering are summarized in Table 2. Several concepts underlying persuasive technology, including personalization, tailoring, and behavioral guidance, are also reflected in digital nudging approaches (Oinas-Kukkonen and Harjumaa, 2009; Weinmann et al., 2016). These conceptual similarities highlight the close relationship between persuasive system design and digital nudging. Building on these foundations, the following section introduces digital nudging and discusses how nudging strategies can be used to support software engineering decisions and practices.

**Table 2:** PSD primary task support principles (Oinas-Kukkonen and Harjumaa, 2009).

Principle	Description
Reduction	Simplify complex tasks into easier steps to lower user effort.
Tunneling	Guide users step-by-step through a process toward the desired outcome.
Tailoring	Adapt system content or functionality to specific user needs or contexts.
Personalization	Customize the system experience to individual users' preferences and characteristics.
Self-monitoring	Allow users to track and reflect on their own behaviors or progress.
Simulation	Provide a representation of how user actions influence outcomes.
Rehearsal	Enable users to practice the target behavior safely or in a simulated environment.

### 2.4 Nudging theory

Nudging is commonly defined as an aspect of choice architecture that influences behavior in a predictable way without restricting available options or significantly altering economic incentives (Thaler and Sunstein, 2008). More generally, nudging seeks to guide or encourage behavior through subtle modifications of the decision environment while preserving users' freedom of choice (Halpern, 2015).

A frequently cited example illustrates how small changes in presentation can influence decisions. For instance, placing healthier food options, such as fruit, in more visible or accessible positions can increase their selection, even when less healthy alternatives remain available (Thaler and Sunstein, 2008).

From a cognitive perspective, nudging is often linked to dual-process theories of decision-making. According to Evans and Stanovich (2013), human behavior can be described in terms of two interacting modes: an *automatic mode*, which is fast and intuitive, and a *reflective mode*, which is slower and more deliberate. Nudging approaches are commonly designed to influence intuitive and automatic decision processes, thereby shaping behavior with relatively little cognitive effort (Kahneman, 2011; Weinmann et al., 2016).

An important characteristic of nudges is that they preserve individual autonomy. Users remain free to ignore the intervention and choose any available alternative (Thaler and Sunstein, 2008). At the same time, the effectiveness and acceptability of nudges depend on contextual factors such as timing, presentation, user goals, and perceived trustworthiness (Weinmann et al., 2016; Meske and Potthoff, 2017). Consequently, poorly designed nudges may be ineffective or may be perceived as intrusive.

Digital nudging is generally regarded as a specific application of persuasive technology in which behavioral influence is achieved through the design of digital choice architectures while preserving users' autonomy (Weinmann et al., 2016; Fogg, 2003). Conventional recommendation systems, in contrast, primarily support decision-making by recommending relevant information or alternatives based on contextual information, historical data, or user preferences (Adomavicius and Tuzhilin, 2005; Ricci et al., 2015). However, modern AI-assisted developer tools increasingly combine recommendation capabilities with behavioral interventions, making the distinction less clear in practice. Accordingly, such systems were considered digital nudging interventions in this review only when their recommendations were explicitly intended to influence developers' behavior or software engineering decisions through the design of the decision environment.

To illustrate these strategies in software engineering contexts, several examples can be found in the reviewed literature.

*Defaults* are increasingly visible in AI-assisted development tools such as GitHub Copilot, where generated code suggestions are presented as the default option that developers may directly accept, modify, or reject (Vaithilingam et al., 2022). The nudging effect stems from making a particular solution immediately available and reducing the effort required to adopt it.

*Framing* is exemplified by the security nudges proposed by Fischer et al. (2019), where developers receive recommendations and warnings that encourage the selection of stronger cryptographic alternatives. The intervention influences decisions by presenting security-relevant information in a way that highlights the benefits of secure choices and the risks associated with insecure alternatives.

*Feedback* is commonly implemented through code review systems. For example, the study by McIntosh et al. (2016) investigates mechanisms that provide developers with information about code quality and review outcomes. Such feedback enables developers to identify and correct issues during development.

*Simplification* can be observed in ArchReco (Sielis et al., 2017), a recommendation system that assists developers in selecting appropriate software design patterns. By narrowing the set of alternatives and providing context-aware suggestions, the system reduces cognitive effort during design decisions. Although ArchReco is fundamentally a recommendation system, it was classified as a digital nudging intervention in this study because it intentionally simplifies architectural decision-making rather than merely presenting ranked alternatives.

*Reminders* are illustrated by the work of Spadini et al. (2020), which investigates interventions that remind developers about secure coding practices and potential vulnerabilities. The reminders are intended to trigger desired actions at appropriate moments within the development workflow.

*Salience* is represented by Tricorder (Sadowski et al., 2018), which highlights code quality and maintainability issues directly within the development process. By making potential problems more visible, the tool increases the likelihood that developers will attend to and address them.

*Social norms* are reflected in collaborative development platforms such as GitHub. The study by Dabbish et al. (2012) shows how the visibility of peer activities, contributions, and community practices can influence individual behavior. The nudging effect is based on social proof and awareness of how others act within the development community.

Finally, *personalization* is increasingly supported by AI- and LLM-based systems. For example, the Grounded Copilot approach investigated by Barke et al. (2023) provides recommendations that are adapted to the developer's current coding context. Such systems tailor behavioral support to individual tasks and situations, thereby increasing their relevance and effectiveness. Within the proposed classification, these context-aware recommendations represent personalized digital nudges because they influence developers' decisions through guidance dynamically adapted to the developer's current task.

Together, these examples illustrate how established digital nudging strategies can be operationalized within software development environments and provide a foundation for the empirical trends discussed later in this article.

## 3 Methodology

The literature search, study selection, and reporting were conducted using a structured workflow informed by established systematic review practices to enhance methodological rigor and reproducibility. Although the review did not follow a formal PRISMA protocol, the study identification and selection workflow reflects the principal stages of systematic study selection while remaining appropriate for a structured narrative review.

This study aims to synthesize representative applications of digital nudging and related persuasive technologies in software development, identify prevailing intervention strategies and research trends, and provide an evidence-based overview rather than exhaustive coverage of the broader literature.

**Figure 1:**
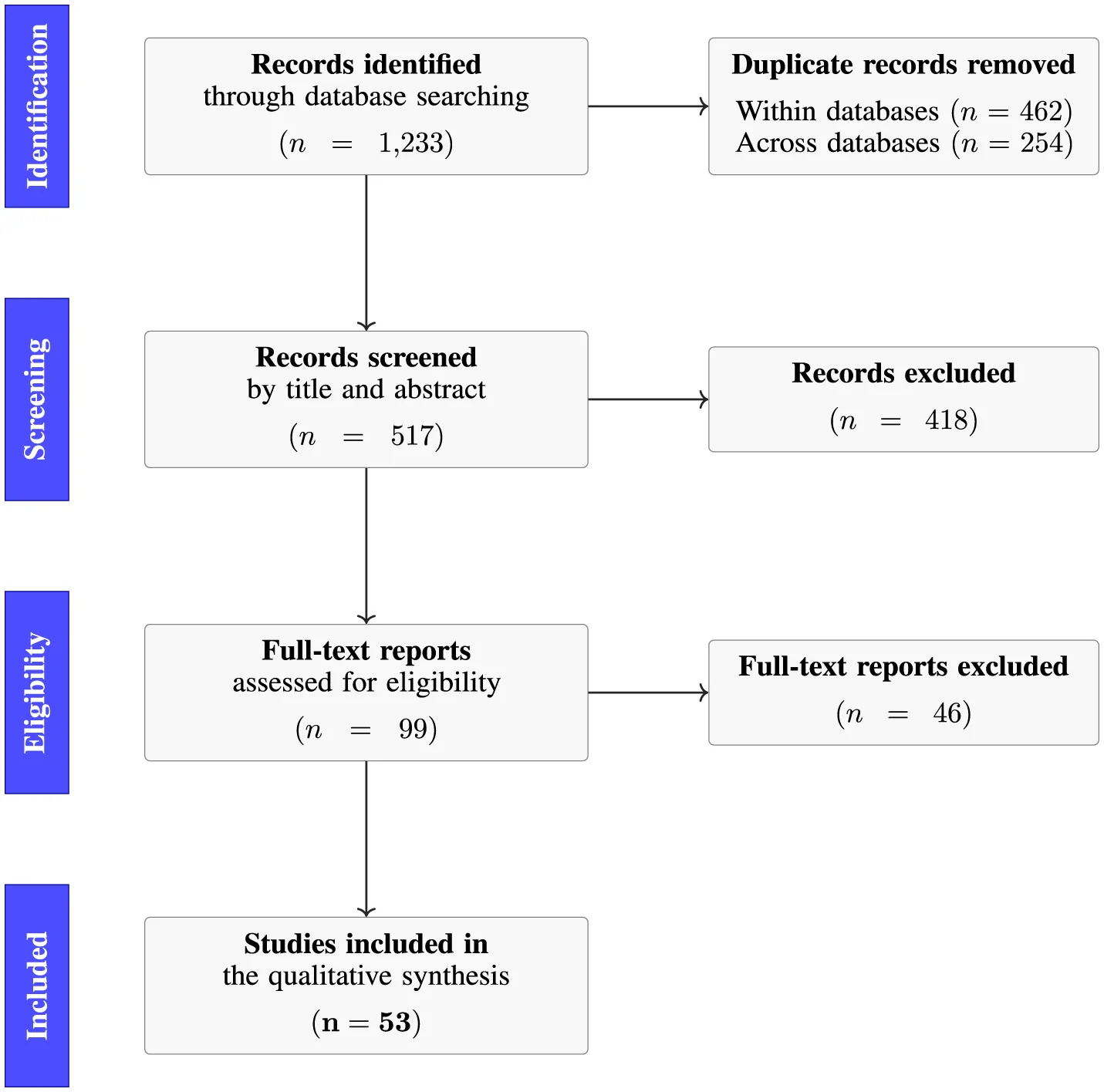
PRISMA-inspired study identification and selection workflow illustrating literature identification, duplicate removal, title and abstract screening, full-text eligibility assessment, and study inclusion.

The methodology comprised five stages: (1) literature identification, (2) duplicate removal, (3) title and abstract screening, (4) full-text eligibility assessment, and (5) data extraction, thematic analysis, and evidence synthesis. Figure 1 summarizes the study identification and selection workflow.

### 3.1 Search strategy

Searches were conducted in major academic databases, including the ACM Digital Library, IEEE Xplore, SpringerLink, ScienceDirect, Taylor & Francis Online, and Google Scholar. In addition to journal articles, peer-reviewed conference proceedings were included, as conferences represent primary dissemination venues in software engineering and human–computer interaction, such as the International Conference on Software Engineering (ICSE), the ACM Conference on Human Factors in Computing Systems (CHI), the International Workshop on Cooperative and Human Aspects of Software Engineering (CHASE), and the International Conference on Computer–Human Interaction Research and Applications (CHIRA).

Search queries combined behavioral intervention terms such as *digital nudging, nudging, persuasive technology, persuasive systems, behavior change*, and *choice architecture* with software engineering terms including *software development, software engineering, software quality, developer tools, plug-ins, bots, technical debt*, and *code review*. Boolean operators were used to identify studies at the intersection of behavioral interventions and software engineering.

To capture recent developments, the search was extended to AI-assisted software development. Additional keywords included *developer behavior, decision support, human–computer interaction, human-centered software engineering, AI-assisted programming, intelligent developer tools, large language models, prompt engineering*, and *human–AI collaboration*.

Search strings were adapted to the syntax and capabilities of each database while preserving the same underlying search concepts. Records retrieved from the individual databases were exported and consolidated prior to duplicate removal.

### 3.2 Study selection and screening

Studies were included if they

1. Examined digital nudging, persuasive mechanisms, or related behavioral interventions,
2. Addressed software engineering or software development,
3. Described or evaluated a developer-facing software artifact intended to influence developer behavior or software engineering decisions (e.g., tools, plug-ins, IDE extensions, dashboards, recommender systems, bots, or AI assistants), and
4. Were published in peer-reviewed venues between 2010 and April 2026.

Studies unrelated to software engineering, non-peer-reviewed publications, studies lacking a developer-facing software artifact, or publications providing insufficient methodological detail were excluded.

The literature search identified **1,233** records across the selected databases. After duplicate removal, **517** unique records remained for title and abstract screening. This screening retained **99** candidate studies for full-text assessment, resulting in **53** primary studies included in the qualitative synthesis.

### 3.3 Data extraction and analysis

Extracted information included publication characteristics, software engineering context, software artifact, intervention strategy, implementation approach, evaluation methodology, AI involvement, and reported outcomes.

The included studies were classified as theoretical contributions, direct nudging interventions, or indirect behavioral influence mechanisms using an iterative multi-label coding approach based on the nudging strategies presented in Table 3. Classification was guided by the behavioral mechanisms explicitly described or implemented by the authors rather than the underlying technical implementation. The coding framework was informed by established digital nudging and persuasive technology literature and refined for software engineering.

**Table 3:** Common digital nudging strategies adapted from Weinmann et al. (2016).

Nudging strategy	Description
Defaults	Pre-selecting options so they become the easiest choice.
Framing	Presenting information in different ways (e.g. gain vs loss framing) to guide decisions.
Feedback	Providing information about actions or their consequences.
Simplification	Reducing complexity and cognitive effort required to make a decision.
Reminders	Prompting users at appropriate moments.
Salience	Highlighting important information to draw attention.
Social norms	Indicating the behavior of others to influence decisions (social proof).
Personalization	Adapting information or options to the individual user.

The synthesized evidence formed the basis for the temporal trend analysis, behavioral heatmaps, comparative evaluation, evidence mapping, and development of the proposed Harmonized Digital Nudging Framework.

## 4 Research landscape (2010–April 2026)

This section examines the evolution of behavioral influence mechanisms, including persuasive technology and digital nudging, in software development from 2010 to April 2026. It is based on the premise that software developers' design decisions may be influenced by cognitive biases and can be improved through structured behavioral support (Kahneman, 2011; Thaler and Sunstein, 2008).

The reviewed studies are analyzed to examine how behavioral influence mechanisms have been applied in software engineering contexts. This analysis is informed by the concept of digital nudging Weinmann et al. (2016) and considers core strategies, including feedback, salience, simplification, and framing (see Table 3).

Table 4 summarizes the evolution of theoretical perspectives that have informed nudging mechanisms in software engineering. The periods reflect major conceptual shifts in the literature, from persuasive technology and behavior change models to digital nudging, context-aware interventions, and, more recently, AI-mediated decision support. The table highlights the dominant theories, associated concepts, and their relevance to software development environments.

**Table 4:** Illustrative evolution of theoretical foundations relevant to persuasive technology and digital nudging.

Period	Theory	Concepts	SE Relevance	References
2010–2015	Persuasive technology and behavior change	Fogg Behavior Model (FBM); Persuasive Systems Design (PSD); Behavior Change Support Systems	Establishes foundational principles for influencing user behavior through feedback, reminders, tailoring, and social support, which informed early behavior-aware software development tools.	Fogg, 2003; Oinas-Kukkonen and Harjumaa, 2009
2015–2020	Digital nudging and choice architecture	Nudge Theory; Digital Nudging; Choice Architecture	Conceptualizes software systems as choice environments in which interface elements such as defaults, framing, and salience may influence users' decision-making.	Thaler and Sunstein, 2008; Weinmann et al., 2016
2020–2024	Context-aware and technology-mediated nudging	Context-aware nudging; adaptive interfaces; behavioral decision support.	Emphasizes the role of timing, context, and automation in delivering behavioral support through software tools and integrated feedback mechanisms.	Caraban et al., 2018; Weinmann et al., 2016
2024–2026	AI-mediated and adaptive decision support	Human–AI interaction; adaptive nudging; AI-assisted decision support	Introduces new opportunities for personalized and dynamically generated behavioral support through large language models, intelligent assistants, and human–AI collaboration.	Amershi et al., 2019; Dwivedi et al., 2023; Weinmann et al., 2016

**Figure 2:**
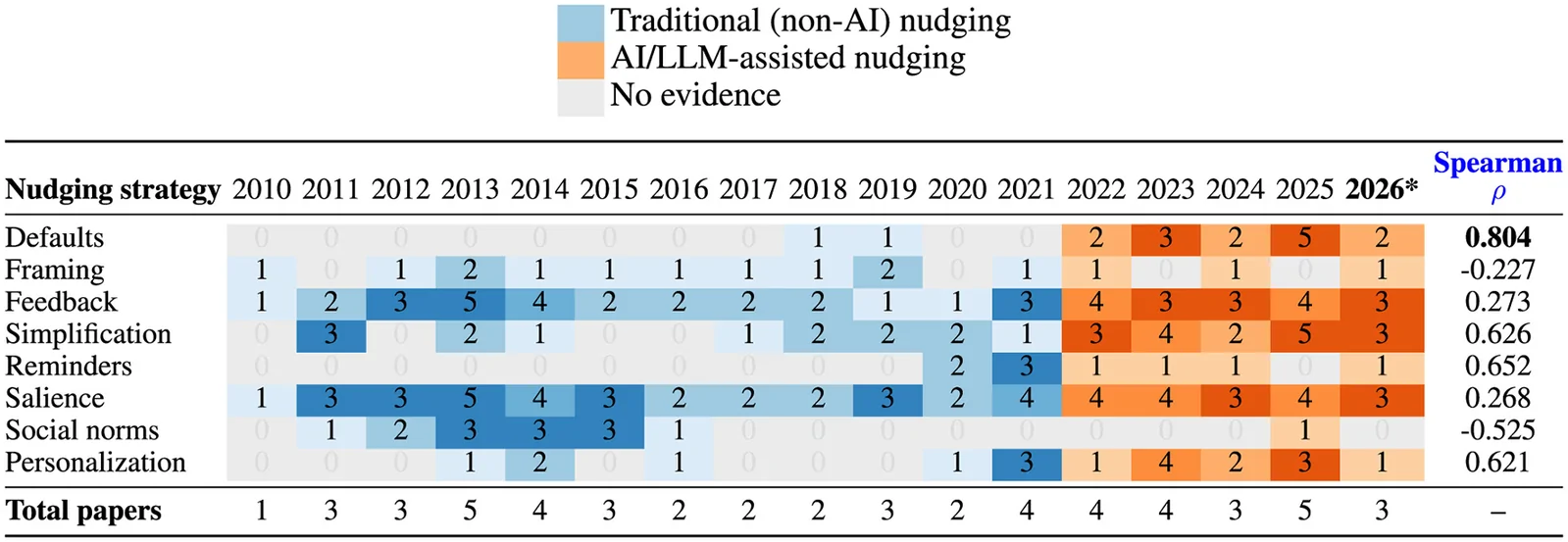
Temporal heatmap of nudging strategies in software engineering, illustrating the evolution from early persuasive and recommendation-oriented approaches toward increasingly adaptive AI- and LLM-assisted behavioral interventions in software development environments. Cells show how frequently each nudging strategy appeared in empirical or tool-based studies within a given year. A multi-label coding scheme was applied; therefore, individual studies may contribute to multiple strategies. The rightmost column reports exploratory Spearman rank correlations between publication year and annual strategy frequency. Gray cells indicate the absence of evidence. The 2026 values represent partial-year data and should be interpreted cautiously.

The table summarizes conceptual perspectives that inform the interpretation of the empirical evidence map shown in Figure 2. The period boundaries are approximate and reflect the increasing prominence of these perspectives in the literature.

Building on the theoretical evolution summarized in Table 4, Figure 2 presents a temporal overview of the 53 empirical studies published between 2010 and April 2026 that describe developer-facing tools or platforms designed to influence developer behavior. The observed temporal patterns reveal a gradual evolution from early rule-based interventions toward increasingly adaptive and AI-assisted behavioral support. Early work is primarily characterized by interventions based on predefined rules and automated feedback (e.g., static analysis checks, warnings, and code quality reports) that identify deviations from coding standards, quality guidelines, or design principles, for example in early work on automated code smell detection (Pribik and Felfernig, 2012). More recent research increasingly incorporates context-aware and adaptive mechanisms (Weinmann et al., 2016).

From 2022 onward, the reviewed literature indicates a growing presence of artificial intelligence (AI) and large language model (LLM)-mediated approaches, enabling more dynamic and personalized forms of behavioral support (Vaithilingam et al., 2022; Barke et al., 2023; Amershi et al., 2019). Recent studies demonstrate growing interest in integrating these mechanisms into software development environments.

Figure 2 shows the temporal distribution of nudging strategies identified in empirical and tool-based software engineering studies. Because the coding categories are not mutually exclusive, annual strategy counts may exceed the number of distinct papers. The strategies follow the taxonomy defined in Table 3. AI/LLM-assisted nudging refers to systems in which behavioral influence is supported by adaptive or generative model outputs, including code suggestions, conversational agents, and IDE-based recommendation systems.

Overall, the results indicate a gradual shift from early persuasive software engineering approaches and context-aware recommendation systems toward increasingly adaptive AI-assisted nudging mechanisms. This transition should not be interpreted as abrupt, as earlier studies had already explored recommendation-oriented, context-aware, and ML-supported developer guidance before the widespread adoption of LLM-based systems (Sielis et al., 2017; Luan et al., 2018; Fischer et al., 2019). Since 2022, however, AI- and LLM-assisted approaches have become increasingly prominent and are frequently combined with established nudging strategies such as feedback, framing, simplification, and personalization. More recent work also extends these approaches to software quality and software design principles, including **SOLID** (Pribik and Felfernig, 2026; Barke et al., 2023).

For transparency and reproducibility, Table A1 in the Appendix summarizes the primary empirical studies included in the temporal heatmap and trend analysis, grouped by publication year.

### 4.1 Trend analysis

To further examine the temporal patterns shown in Figure 2, exploratory Spearman rank correlations were computed between publication year and the annual frequency of each nudging strategy. Positive coefficients indicate increasing presence over time, whereas negative coefficients indicate decreasing presence. The resulting coefficients are reported directly in Figure 2. These coefficients are interpreted descriptively and exploratorily, given the relatively small number of studies, the multi-label coding scheme, and the partial-year coverage for 2026.

The analysis reveals several recurring patterns. Feedback-based mechanisms appear consistently across all periods, while strategies such as social norms are more frequently observed in earlier work. In contrast, personalization-related approaches are more commonly reported in recent studies, particularly in connection with AI/LLM-based systems.

The exploratory correlations further differentiate these temporal patterns. Defaults exhibit the strongest positive temporal association (ρ = 0.804). Positive associations are also observed for reminders (ρ = 0.652), simplification (ρ = 0.626), and personalization (ρ = 0.621), consistent with their greater representation in recent years. By contrast, social norms show a negative temporal association (ρ = −0.525), reflecting their greater prominence in earlier studies. Feedback (ρ = 0.273), salience (ρ = 0.268), and framing (ρ = −0.227) exhibit weaker temporal associations and no comparable monotonic pattern.

In addition, some strategies appear to be used in combination. For example, feedback mechanisms are often accompanied by elements of personalization, while framing and salience are sometimes jointly applied to influence how information is presented and perceived.

Taken together, these patterns demonstrate an increasing emphasis on context-sensitive and individualized forms of developer support. Building on these findings, three broader research trends can be identified.

First, there is a shift toward more process-oriented and fine-grained interventions, where nudging mechanisms increasingly target decisions during development rather than outcomes after completion. This is reflected in the growing use of real-time suggestions and interactive guidance.

Second, there is a transition from rule-based approaches toward data-driven and adaptive mechanisms, often supported by AI/LLM-based systems that enable context-aware and personalized guidance.

Third, nudging mechanisms are increasingly integrated directly into development environments and extended toward higher-level concerns, including support for software design principles. There are also indications of emerging approaches that explicitly target design principles, such as guiding developers toward adherence to **SOLID**.

However, the effectiveness of these strategies is likely dependent on their specific implementation. For instance, salience-based interventions may lead to alert fatigue when overused Weinmann et al. (2016), while default-based approaches may raise questions regarding developer autonomy (Thaler and Sunstein, 2008).

**Figure 3:**
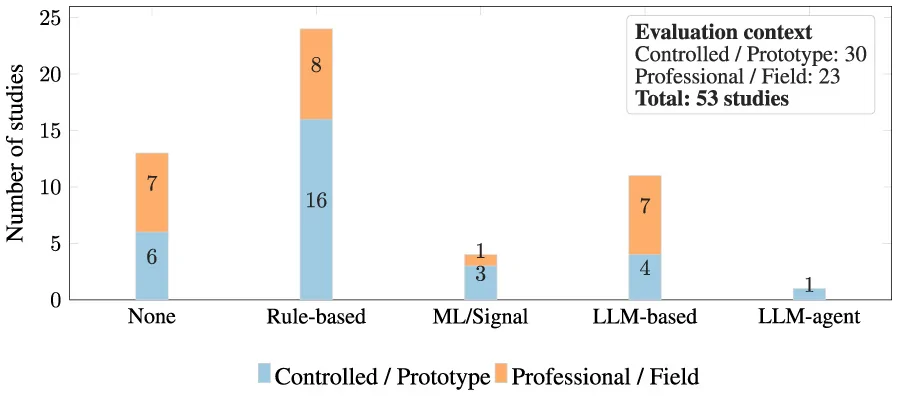
Distribution of reviewed studies by dominant technical component and evaluation context. The categories distinguish studies with no explicit technical mechanism (None), rule-based or heuristic systems, machine learning (ML) or signal-analysis systems, large language model (LLM)-based assistants, and LLM-based agents. “Professional / Field” denotes studies involving professional developers, industrial settings, field deployments, or other realistic development contexts.

Figure 3 provides a complementary view of the reviewed studies by classifying them according to their dominant technical component and evaluation context. The distribution reveals that rule-based systems remain the most frequently studied category, while large language model (LLM)-based approaches have emerged more recently. The figure also shows that most studies were evaluated in controlled or prototype settings, with comparatively fewer studies involving professional developers or field deployments.

Despite these developments, there are indications that the integration of multiple nudging strategies into cohesive, developer-centered tools remains limited. Similarly, explicit support for software design principles has only recently begun to emerge in the literature.

Finally, it should be noted that the most recent period (2025–April 2026) reflects early-stage developments, and the observed increase in AI-mediated approaches should be interpreted as an emerging trend rather than a fully established pattern.

## 5 Toward harmonized digital nudging in software development

### 5.1 Motivation for harmonization

The preceding analysis demonstrates that digital nudging in software development has evolved toward more adaptive, context-aware, and AI-mediated approaches. At the same time, existing work remains fragmented, with individual studies focusing on specific nudging strategies, tools, or development contexts (Weinmann et al., 2016; Meske and Potthoff, 2017). This fragmentation makes it difficult to systematically design and integrate nudging mechanisms that provide consistent and scalable support across development activities.

Building on the trends identified in the literature review and temporal analysis, these observations highlight the need for a more harmonized perspective, in which multiple nudging strategies are systematically combined and aligned with developer tasks, contexts, and goals.

### 5.2 Conceptualization of harmonized digital nudging

In this article, harmonized digital nudging refers to the structured integration and alignment of multiple nudging strategies within software development environments to provide consistent, context-aware, and adaptive decision support (Thaler and Sunstein, 2008; Weinmann et al., 2016).

Rather than relying on isolated interventions, harmonized digital nudging combines multiple mechanisms, such as feedback, framing, and personalization, to provide coordinated support that is aligned with both the development context and the cognitive characteristics of software developers. A central principle of this approach is that behavioral interventions should be designed in a manner that preserves user autonomy and supports transparent decision-making (Thaler and Sunstein, 2008; Meske and Potthoff, 2017).

The increasing presence of AI- and LLM-based development tools in recent software engineering research, as reflected in the trend analysis (Figure 2), creates opportunities for systems that dynamically combine and adapt nudging strategies to individual developers, development tasks, and project contexts. In this sense, harmonized digital nudging offers a conceptual perspective for understanding how behavioral support mechanisms can be systematically coordinated within software development workflows.

### 5.3 Design dimensions for harmonized nudging

The following design dimensions were derived from recurring themes identified in the reviewed literature on persuasive technology, digital nudging, and adaptive decision support (Weinmann et al., 2016; Meske and Potthoff, 2017). Collectively, these dimensions characterize the proposed concept of *harmonized digital nudging*, which integrates behavioral, contextual, technical, and ethical considerations to support software engineering decision-making. Rather than representing independent design principles, the dimensions are intended to complement one another and should be understood as an integrated conceptual framework.

Although each of the 11 design dimensions addresses a distinct aspect of digital nudging, they are intended to operate in combination throughout the design and delivery of behavioral interventions. Developer context, task characteristics, and skill level influence the selection and adaptation of appropriate nudging strategies, whereas transparency and responsible AI constrain their implementation to preserve developer autonomy and trust. The eleven design dimensions comprising the proposed framework are described below.

**Strategy integration:** multiple nudging strategies should be combined in a complementary manner, for example by integrating feedback with personalization or framing, rather than applying them in isolation (Caraban et al., 2019; Meske and Potthoff, 2017).

**Context awareness:** nudging mechanisms should be adapted to the specific development phase (e.g., design, implementation, or code review) and the task at hand (Caraban et al., 2018; Fischer and Grossklags, 2022).

**Timing alignment:** the timing of nudges is critical, as interventions should be delivered at moments where they can effectively influence decisions without unnecessarily interrupting developer workflows (Fogg, 2009; Fischer and Grossklags, 2022).

**Adaptive personalization:** AI/LLM-based systems create new opportunities for nudging mechanisms to adapt dynamically to developer behavior, preferences, and experience levels (Santilli et al., 2025; Ganapini et al., 2023).

**Skill awareness:** to support long-term effectiveness, nudging mechanisms should be aligned with developers' skill levels and domain knowledge. This can help prevent cognitive overload for less experienced developers while reducing redundant guidance for more experienced users (Caraban et al., 2018; Santilli et al., 2025).

**Tool integration:** nudging mechanisms should be embedded directly into development tools to provide continuous and minimally intrusive support (Pribik and Felfernig, 2012; Fischer and Grossklags, 2022).

**Transparent nudging:** developers should be aware that nudging mechanisms are present and understand their purpose. Transparent design can increase trust and acceptance and helps ensure that nudging supports decision-making without being perceived as manipulative (Thaler and Sunstein, 2008; Meske and Potthoff, 2017).

**Social feedback:** providing aggregated or anonymized insights into the behavior of other developers or teams can support learning and motivation, for example by highlighting alternative design approaches or best practices (Egel et al., 2011; Dabbish et al., 2012). Such mechanisms should be carefully designed to avoid negative effects such as excessive competition or pressure.

**Responsible AI:** in AI- and LLM-assisted development environments, nudging mechanisms increasingly rely on automatically generated recommendations and code suggestions. This introduces challenges related to the direct adoption of generated artifacts, including potential risks in security, maintainability, correctness, and licensing. Developers should therefore be encouraged to critically evaluate AI-generated suggestions rather than adopting them unreflectively (Amershi et al., 2019; Hassan et al., 2024).

**Quality alignment:** nudging mechanisms can be more effective and better accepted when they are explicitly linked to preferences, thresholds, or quality targets defined by developers or development teams. Examples include thresholds for code metrics, security indicators, or benchmark values derived from prior projects or comparable teams. Aligning interventions with such user-defined criteria can help reduce perceptions of external control and position nudging as a form of delegated decision support rather than unsolicited interference (Thaler and Sunstein, 2008; Meske and Potthoff, 2017).

**Consequence awareness:** the effectiveness of nudges can be strengthened when systems communicate the potential consequences of accepting or ignoring a recommendation. Presenting concise explanations of likely outcomes can support more informed decision-making and make longer-term implications more salient (Amershi et al., 2019; Weinmann et al., 2016).

**Figure 4:**
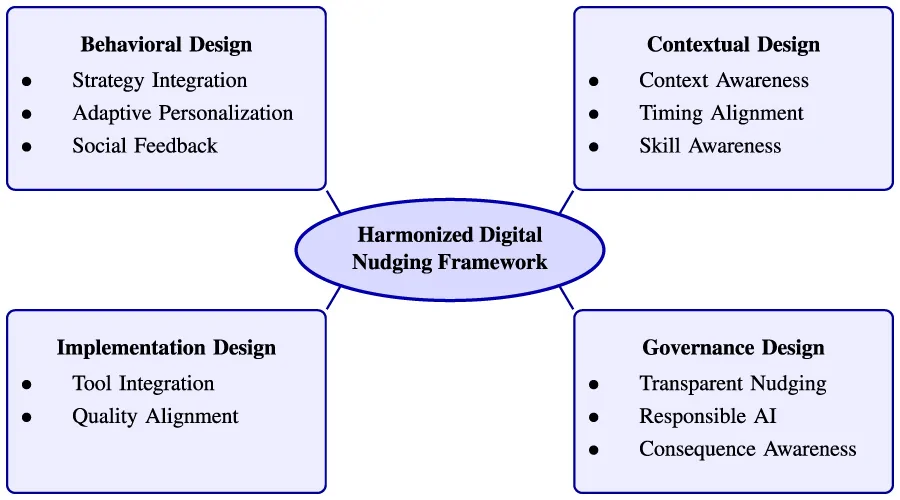
Conceptual organization of the proposed harmonized digital nudging framework. The 11 design dimensions are organized into four complementary design perspectives: Behavioral Design, Contextual Design, Implementation Design, and Governance Design. Together, these perspectives provide an integrated framework for designing behavioral support in software development environments.

The proposed harmonized digital nudging Framework synthesizes the eleven design dimensions derived from the reviewed literature into four complementary design perspectives. Rather than representing independent recommendations, the framework conceptualizes behavioral support in software development as the integration of behavioral, contextual, implementation, and governance considerations. As illustrated in Figure 4, each perspective captures a distinct aspect of harmonized digital nudging while remaining closely interconnected with the others.

Figure 4 synthesizes the proposed harmonized digital nudging Framework by organizing the eleven design dimensions into four complementary design perspectives: Behavioral Design, Contextual Design, Implementation Design, and Governance Design. Together, these perspectives provide a structured view of how behavioral support can be designed within software development environments.

Each design perspective addresses a distinct aspect of developer support. Behavioral Design defines which nudging strategies are applied, Contextual Design determines when and under which conditions interventions should be delivered, Implementation Design describes how they are embedded within software development environments, and Governance Design ensures that behavioral support remains transparent, trustworthy, and aligned with responsible AI principles.

Although organized into separate design perspectives, the eleven design dimensions are intended to operate in combination. For example, the selection of behavioral strategies is guided by contextual factors such as the developer's task, timing, and skill level, implemented through appropriate tool support and quality-related mechanisms, and constrained by governance considerations including transparency, responsible AI, and consequence awareness (Caraban et al., 2018; Meske and Potthoff, 2017; Amershi et al., 2019). Accordingly, the proposed framework should not be interpreted as a collection of independent dimensions but as a network of mutually reinforcing design considerations in which decisions within one perspective shape and constrain the others. Effective digital nudging therefore emerges from the integrated application of dimensions across all four design perspectives rather than from any individual dimension in isolation.

Derived from the reviewed literature, the proposed harmonized digital nudging Framework provides a structured conceptual foundation for future empirical research and the design of AI-assisted software engineering environments. Rather than prescribing a fixed implementation process, it offers a flexible design perspective in which different dimensions become more or less prominent depending on the software engineering context.

## 6 Illustrative example: AI-driven nudging for SOLID compliance

To illustrate the proposed harmonized digital nudging framework, we present a prototype AI-assisted plugin that integrates multiple forms of developer support within an integrated development environment. The prototype extends our preliminary work reported by Pribik and Felfernig (2012, 2026) and Pribik et al. (2026), demonstrating how multiple harmonized nudging dimensions can be combined to provide context-aware, AI-assisted guidance during software development.

The plugin analyzes code changes and evaluates potential deviations from selected **SOLID** principles using an LLM-based analysis. When a potential violation of the Single Responsibility Principle is detected, developers receive a contextual notification together with a concise explanation of the issue and possible refactoring alternatives generated by the underlying AI system.

**Figure 5:**
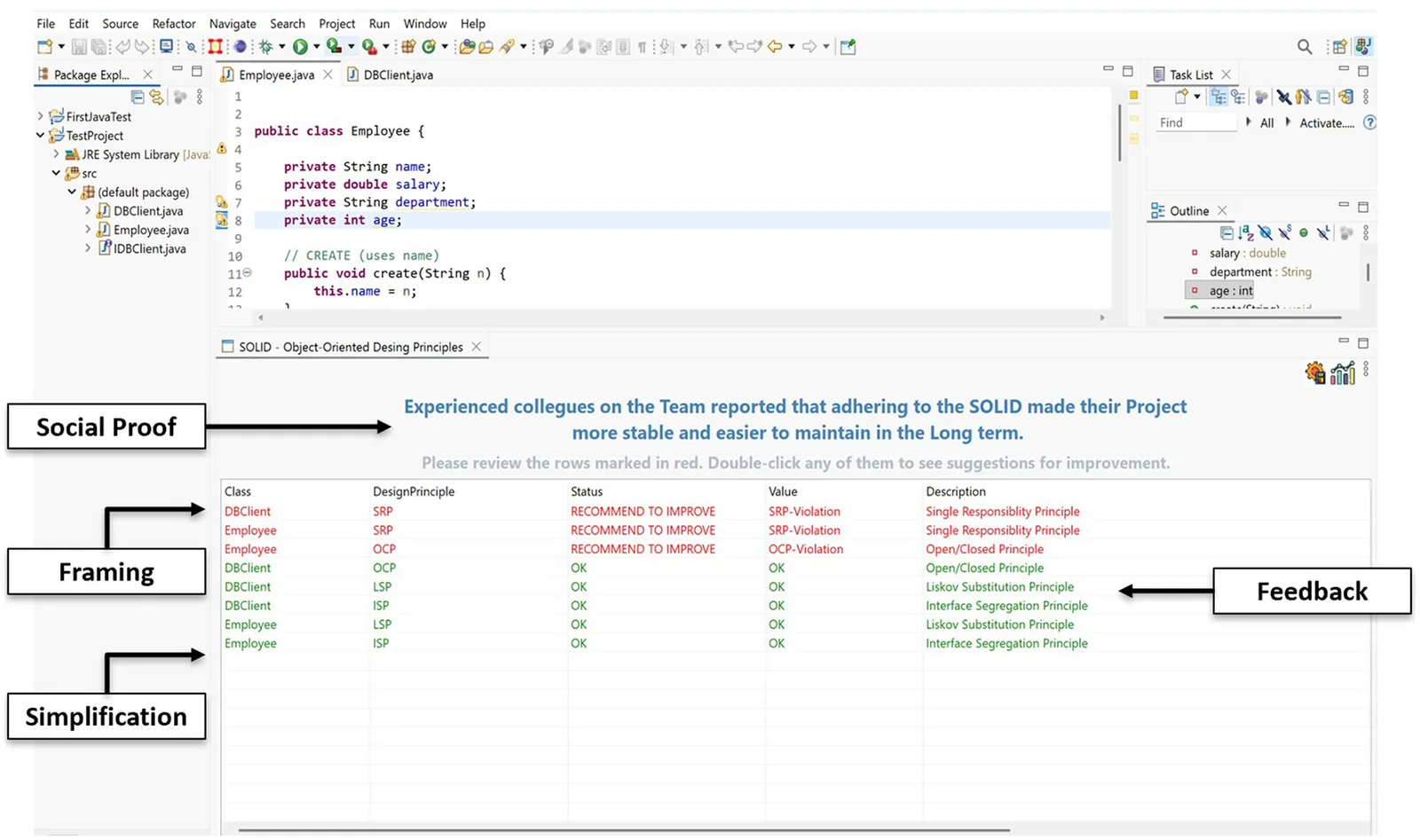
Illustrative example of an AI-assisted harmonized nudging environment supporting adherence to **SOLID** principles through recommendations, quality metrics, and transparent developer guidance.

Figure 5 shows how the prototype combines notifications, recommendations, consequence-aware explanations, and configurable quality thresholds to provide context-sensitive guidance during development activities. From the perspective of the proposed harmonized nudging dimensions, the plugin reflects **Tool Integration** through its embedding within the development environment, **Timing Alignment** through real-time feedback, **Quality Alignment** through the use of software metrics and configurable quality criteria, and **Strategy Integration** through the combination of complementary nudging mechanisms. Specifically, the prototype combines feedback to increase awareness of potential design issues, framing to highlight the benefits of applying SOLID principles, simplification to reduce the effort required to identify and implement suitable refactorings, and social proof to reinforce recommended practices through examples of successful application.

Explanatory feedback and optional recommendations further support **Transparent Nudging**, **Consequence Awareness**, and **Responsible AI**. Each recommendation provides information about the detected issue, the underlying reasoning, and the expected benefits and trade-offs of the proposed solution, thereby supporting informed decision-making while preserving the developer's ability to ignore or override suggestions.

At the same time, such approaches raise challenges related to transparency, user acceptance, and the responsible use of AI-generated recommendations, which are recognized as important considerations in human-centered AI research (Amershi et al., 2019). Developers should remain aware of the presence and purpose of nudging mechanisms, consistent with the principle that nudges should preserve user autonomy and freedom of choice (Thaler and Sunstein, 2008).

The example should therefore be viewed as an exploratory prototype illustrating how harmonized digital nudging concepts may be applied in software development environments. While an initial evaluation with six IT experts, reported in Pribik and Felfernig (2026), provided insights into the prototype's perceived usefulness and applicability, broader empirical studies are needed to assess its effectiveness in industrial practice.

## 7 Open research challenges

The preceding analysis highlights growing interest in integrating digital nudging into software development environments. However, the effective design and integration of such mechanisms remain challenging and require careful consideration of both technical and human factors. Prior research suggests that successful integration depends not only on technical implementation but also on alignment with user needs and development contexts (Fischer and Grossklags, 2022).

As shown by the reviewed studies, existing frameworks propose structured approaches for defining nudging objectives and evaluating their effectiveness (Schneider et al., 2018). These frameworks emphasize factors such as personalization, contextual awareness, timing, and environmental conditions. Despite these advances, the review identified several research challenges that remain insufficiently addressed in software engineering.

**Strategy integration:** identifying suitable combinations of nudging strategies for software development tasks remains an open challenge. Prior work on digital nudging and persuasive system design highlights the complexity of combining multiple intervention mechanisms within interactive environments (Caraban et al., 2018, 2019). Limited evidence is available on how mechanisms such as feedback, framing, and personalization interact within development environments. Further empirical studies are needed to examine their potential effects on developer behavior and software quality.

**Developer diversity:** developers differ in experience, skills, motivations, and working contexts. Designing nudging mechanisms that are appropriate across such diverse populations remains challenging. Existing studies often rely on relatively small or controlled samples, which may limit the transferability of findings to real-world software development environments (Chattopadhyay et al., 2022).

**Impact evaluation:** quantifying the effects of nudging on software development processes remains challenging. Standardized metrics and evaluation methodologies for assessing nudging interventions in software engineering contexts remain limited. Longitudinal studies may be particularly valuable for assessing sustained effects over time (Mertens et al., 2022).

**Interface design:** the effectiveness of nudging is closely linked to how nudges are presented within development tools. Further research is needed to examine how interface design, visualization, and interaction techniques influence whether nudges are perceived as supportive rather than intrusive (Hummel and Maedche, 2019).

Taken together, these challenges reinforce the need for harmonized approaches that systematically integrate behavioral, contextual, technical, and ethical considerations when designing nudging mechanisms for software development. AI- and LLM-based systems also create new opportunities to deliver personalized and context-sensitive interventions directly within development environments. Such approaches may provide additional support for software quality and adherence to established design principles, such as **SOLID**.

## 8 Conclusion

Software development involves complex decision-making processes that may be influenced by cognitive biases. This article examined the role of persuasive technology and digital nudging in supporting developer decision-making, with particular attention to how such mechanisms have been discussed and applied in software engineering contexts.

The review shows that nudging approaches are increasingly being integrated into development environments, with recent work reflecting growing interest in adaptive, context-aware, and AI-mediated mechanisms. Personalized and real-time support may offer additional opportunities to assist developers during the development process.

Building on these observations, this article introduced the concept of harmonized digital nudging, defined as the structured integration of multiple nudging strategies aligned with development contexts and developer needs. This concept is intended to provide an integrated conceptual perspective for designing more consistent and context-aware behavioral support within development workflows.

Within this harmonized perspective, AI- and LLM-based nudging mechanisms offer opportunities to support software quality, including adherence to design principles such as SOLID. These developments suggest potential opportunities for integrating behavioral support directly into development environments while preserving developer autonomy and transparency.

At the same time, several research challenges remain. The selection and combination of appropriate nudging strategies, their timing and presentation, and their longer-term effects on developer behavior require further investigation. In addition, questions related to transparency, user acceptance, and the influence of cognitive biases in industrial development settings remain open. Future research may extend the proposed harmonized digital nudging perspective by developing formal computational models that support adaptive decision-making through balancing intervention effectiveness, developer autonomy, cognitive load, long-term acceptance, and organizational governance constraints. Such models could provide a foundation for AI-assisted developer support systems that adapt the selection, timing, and personalization of nudging strategies while respecting ethical and organizational requirements.

Overall, the findings support the view that digital nudging represents a potentially valuable area for supporting software development. Continued empirical and design-oriented research will be important for evaluating, refining, and operationalizing harmonized nudging approaches in practical software engineering environments.

## Appendix

### Studies included in the heatmap and trend analysis

**Table A1:** Chronological overview of the primary empirical studies underlying the temporal heatmap (Figure 2) and the associated trend analysis, grouped by publication year.

Year	Referenced papers in heatmap (Figure 2)
2010	Steinparz et al., 2010.
2011	Parnin and Rugaber, 2011; Parnin and DeLine, 2011; Egel et al., 2011.
2012	Pribik and Felfernig, 2012; Dabbish et al., 2012; Domínguez et al., 2012.
2013	Balachandran, 2013; Dubois and Tamburrelli, 2013; Grant and Betts, 2013; Singer et al., 2013; Czerwonka et al., 2013.
2014	Fritz et al., 2014; Storey et al., 2014; Tsay et al., 2014; Stikkolorum et al., 2014.
2015	Vasilescu et al., 2015; Witschey et al., 2015; Prause and Jarke, 2015.
2016	McIntosh et al., 2016; Gasparic et al., 2016.
2017	Tufano et al., 2017; Sielis et al., 2017.
2018	Sadowski et al., 2018; Luan et al., 2018.
2019	Wessel et al., 2019; Acar et al., 2019; Fischer et al., 2019.
2020	Spadini et al., 2020; Maddila et al., 2020.
2021	Brown and Parnin, 2021; Tahaei et al., 2021; Zavaleta Bernuy et al., 2021; Pereira and D́ıaz, 2021.
2022	Haug et al., 2022; Vaithilingam et al., 2022; Pearce et al., 2022; Shan et al., 2022.
2023	Barke et al., 2023; Peng et al., 2023; Mastropaolo et al., 2023; Bobadilla et al., 2023.
2024	Alkadhi et al., 2024; Moradi Dakhel et al., 2024; Zhang et al., 2024.
2025	Becker et al., 2025; Kumar et al., 2025; Afroz et al., 2025; Wang and Duan, 2025; Alami et al., 2025.
2026	Pribik and Felfernig, 2026; Merler et al., 2026; Chen et al., 2026.

The selection follows the applied inclusion criteria and focuses on the highly selective core literature (maximum six studies per year). Conceptual and secondary studies used exclusively for theoretical background are not included.
